# A further critique of the analytic strategy of adjusting for covariates to identify biologic mediation

**DOI:** 10.1186/1742-5573-1-4

**Published:** 2004-10-08

**Authors:** Jay S Kaufman, Richard F MacLehose, Sol Kaufman

**Affiliations:** 1 Department of Epidemiology, University of North Carolina School of Public Health, Chapel Hill, NC 27599-7435 USA; 2 Department of Otolaryngology, University at Buffalo, 3435 Main Street, Buffalo NY 14214 USA

**Keywords:** effect decomposition, causality, confounding, counterfactual models, bias

## Abstract

**Background:**

Epidemiologic research is often devoted to etiologic investigation, and so techniques that may facilitate mechanistic inferences are attractive. Some of these techniques rely on rigid and/or unrealistic assumptions, making the biologic inferences tenuous. The methodology investigated here is *effect decomposition*: the contrast between effect measures estimated with and without adjustment for one or more variables hypothesized to lie on the pathway through which the exposure exerts its effect. This contrast is typically used to distinguish the exposure's *indirect *effect, through the specified intermediate variables, from its *direct *effect, transmitted via pathways that do not involve the specified intermediates.

**Methods:**

We apply a causal framework based on latent potential response types to describe the limitations inherent in effect decomposition analysis. For simplicity, we assume three measured binary variables with monotonic effects and randomized exposure, and use difference contrasts as measures of causal effect. Previous authors showed that confounding between intermediate and the outcome threatens the validity of the decomposition strategy, even if exposure is randomized. We define exchangeability conditions for absence of confounding of causal effects of exposure and intermediate, and generate two example populations in which the no-confounding conditions are satisfied. In one population we impose an additional prohibition against unit-level interaction (synergism). We evaluate the performance of the decomposition strategy against true values of the causal effects, as defined by the proportions of latent potential response types in the two populations.

**Results:**

We demonstrate that even when there is no confounding, partition of the total effect into direct and indirect effects is not reliably valid. Decomposition is valid only with the additional restriction that the population contain no units in which exposure and intermediate interact to cause the outcome. This restriction implies homogeneity of causal effects across strata of the intermediate.

**Conclusions:**

Reliable effect decomposition requires not only absence of confounding, but also absence of unit-level interaction and use of linear contrasts as measures of causal effect. Epidemiologists should be wary of etiologic inference based on adjusting for intermediates, especially when using ratio effect measures or when absence of interacting potential response types cannot be confidently asserted.

## 1. Introduction

A large portion of epidemiologic research is devoted to etiologic investigation, and so techniques that may facilitate mechanistic inferences are sought by researchers and are applied frequently in their work. Unfortunately, some of these techniques have been found to provide far more ambiguous evidence on which to base mechanistic conclusions than was first believed. For example, analysis of patterns of joint effects has been proposed as a means of identifying causal structure [1], but simple counterexamples show that in general the underlying etiologic model cannot be readily identified[2]. Typically, some method is proposed under a sound theoretical argument in a specific analytic setting, but this method is subsequently applied in a more general context in which those specific theoretical conditions no longer hold. For example, Greenland and Poole[3] provide a rational justification for deviation from additive joint effects as the benchmark for identifying mechanistic interaction between two factors [[4], pp. 332–339]. But this argument is not generally valid as is often assumed; it doesn't hold for all causal structures and target populations[5].

The list of such untenable overgeneralizations in epidemiologic practice is surely large and varied, and has led to any number of false conclusions and misunderstandings. We describe here one particular epidemiologic technique that is applied frequently in practice, and yet is invalid in all but a surprisingly narrow range of circumstances. It is a remarkable example in that the analytic strategy is exceedingly common, and yet is described infrequently in epidemiologic texts or methodologic articles. The few textbook citations that do exist provide no formal justification, and therefore there is little guidance available from within the sources in our field to guide users and warn them of important limitations of this approach. This situation motivates the present article, in which we will show that although widely applied, this analytic approach is almost never justifiable on the basis of reasonable assumptions about the data.

The methodologic approach of interest in this article is the decomposition of effects purportedly accomplished by contrasting two adjusted effect estimates for the exposure of interest: an estimate adjusted for potential confounders, and an estimate adjusted for the same set of potential confounders plus one or more additional variables hypothesized to be causal intermediates, i.e., to lie on pathway(s) through which the exposure exerts its effect. This contrast is then typically used to distinguish the exposure's *indirect *effect, through the specified intermediate variables, from its *direct *effect, transmitted via pathways that do not involve the specified intermediate variables. If control of hypothetical causal intermediates greatly attenuates an exposure's estimated effect, it is generally inferred that the exposure's effect is mediated primarily through pathways involving these quantities; a small degree of attenuation is interpreted as evidence that other pathways predominate. These mechanistic inferences then inform policy recommendations concerning the utility of potential interventions. Although this effect decomposition approach is quite common in the epidemiologic literature, its general validity has not been adequately investigated.

This analytic strategy for effect decomposition in epidemiologic research is recommended by Susser [[6], pp. 121–124], and more recently by Szklo & Nieto [[7], pp. 184–187]. The latter authors quantify the degree of mediation as follows:

" The degree to which a given mechanism...explains the relationship of interest is given by the comparison of adjusted (A) and unadjusted (U) measures of association (e.g., a relative risk, RR). This comparison can be made using the ratio of the unadjusted RRs, RR_U_/RR_A_, or the percent excess risk explained by the variables adjusted for:  ".

Calculations similar to this "% Excess Risk Explained" are the most common framework for describing the effect decomposition analysis in epidemiologic research.

For example, a study by Heck and Pamuk investigated the relation between education and postmenopausal breast cancer incidence using data from the National Health and Nutrition Examination Survey I Epidemiologic Follow-up Study[8]. Proportional hazards modeling was used to estimate the relation between breast cancer incidence and education level. The authors then reported that reproductive factors including nulliparity were found to mediate this relation. This assertion was based on the observation that adjustment for these factors reduced the magnitude of the positive relation between education level and risk of postmenopausal breast cancer. Furthermore, because the association between exposure and outcome was no longer statistically significant after adjustment for the putative mediators, the authors concluded that "the association between higher education and increased risk of breast cancer appears to be largely explained by differences in the known risk factors for breast cancer" [[8], p. 366]. This methodology is commonly applied, and therefore there are many similar examples in the published literature. On the basis of this approach numerous authors have made many similar mechanistic claims about mediation, for example, that blood pressure mediates the causal relation between homocysteine and cardiovascular risk[9], that behavioral risk factors mediate the causal relation between hostility and incident myocardial infarction[10], and that the protective effect of gene CCR5 heterozygosity on clinical AIDS occurrence is completely mediated through an effect on CD4 cell count[11]. The results of decomposition analyses are also frequently used to anticipate the impact of a potential intervention or policy related to the intermediate variable(s). For example, Lantz and colleagues adjusted for several measured behavioral intermediates in assessing the relation between income and mortality[12]. They noted that even after adjustment for these measured intermediates "the risk of dying was still significantly elevated for the lowest-income group (hazard rate ratio, 2.77; 95% CI, 1.74–4.42)..." and on this basis they offered the conclusion that "socioeconomic differences in mortality ...would persist even with improved health behaviors among the disadvantaged." [[12], p. 1703]

In a seminal article on the topic, Robins & Greenland[13] employed a causal framework based on latent potential response types in order to describe the limitations inherent in the effect decomposition analysis. Subsequent authors have for the most part focused on the Robins & Greenland finding that direct effect estimates, decomposed from the total effect by adjusting for an intermediate, may be biased if there is unmeasured confounding between the intermediate and the outcome [14-16]. As Robins & Greenland showed and these later authors reiterated, the decomposition strategy may fail even when the total effect is unconfounded. While this consideration is important, this is not our concern in the present discussion. Rather, we will show that even when there is no confounding of any relevant causal effect, the decomposition strategy will still generally fail, in the sense that a contrast such as that described above as the "% Excess Risk Explained" will fail to provide an unbiased estimate of the proportion of the causal effect that is relayed through the intermediate.

Our critique would appear to contradict standard practice in the social sciences, in which decomposition analysis is also commonly applied[17,18]. We suggest two explanations for this state of affairs. The first is that the development of the decomposition methodology by Wright[19] and other pioneering social science statisticians did not make use of an explicitly casual framework, but rather was derived algebraically from linear regression theory. One consequence is that the causal assumptions necessary for the model to be substantively meaningful were not readily apparent until the advent of a notational system for potential outcomes[20]. Secondly, we suggest that this is another example in which unwitting users have extrapolated a technique beyond the strictly defined original set of assumptions without assessing the impact of this extrapolation on the validity of the estimation. In this case, assumptions imposed in the original development of the decomposition methodology involved additivity of effects and linear contrast measures, neither of which are typical of the analysis of discrete events, such as occurrence of disease. Epidemiologists as well as others have generally been remiss in failing to attend to these crucial assumptions when applying these techniques more broadly. However, as we will describe, the causal assumptions required for the validity of the decomposition method are not verifiable from observed data, and furthermore are unrelated to any typical substantive knowledge. It may therefore be essentially impossible to apply this methodology with any confidence in a real-world analysis of data.

## 2. Framework, Notation and Causal Structure

**Figure 1:**
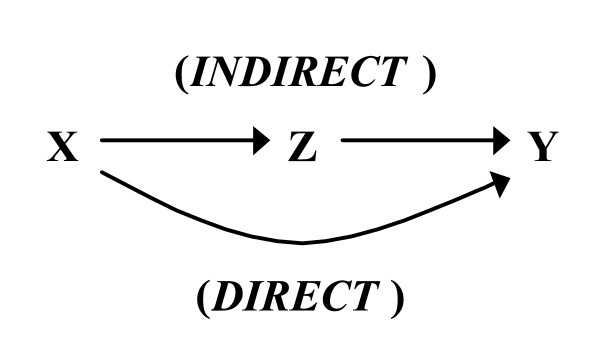
Decomposition of Total Effect of X on Y into Direct and Indirect Effects. The total average causal effect (ACE) of X on Y is achieved through two pathways, one which is termed "indirect'' because it operates through measured intermediate variable Z, and another that is termed "direct'' because it operates through no measured intermediates.

For clarity, we limit our exposition to the simplest possible decomposition problem, which is the structure that includes three measured binary variables and sample size sufficiently large to justify the assumption of zero sampling error (see endnote 1). The three variables are designated as X, Y and Z. The causal relationships between these nodes are described by the directed acyclic graph (DAG)[21] in Figure 1. X is a randomly assigned (i.e., exogenous) treatment and therefore there are no arrowheads terminating at this node in the graph. X takes the value of 1 if treated, 0 otherwise. Y equals 1 if the outcome occurs, and 0 otherwise. Z takes the value of 1 if the intermediate occurs, and 0 otherwise, and like X is manipulable (i.e., may be fixed through external intervention to take either level). The framework adopted here is a deterministic counterfactual model in which each individual unit in the population is assumed to have a fixed potential response to each possible input pattern at each endogenous node of the DAG. As such, the observed data reveal only a subset of these fixed potential responses. We also assume that the potential responses of each unit do not depend on the treatments assigned the other units, which is referred to by Rubin as the "stable-unit-treatment-value assumption" (SUTVA)[22].

The potential response variable for unit *u *at node Z is denoted by Z_ux _where index *u *identifies the individual unit and index x specifies the X value factually or counterfactually experienced by that unit. Given the deterministic model at the individual unit level, there are four possible patterns of response Z_ux _to input x that unit *u *can exhibit, and these have received various appellations in the literature, such as "doomed" for Z_ux_= 1 regardless of x, "causal" for Z_ux_= x, "preventive" for Z_ux_= 1-x, and "immune" for Z_ux _= 0 regardless of x[23]. These four patterns may be represented by potential response type index values of 1, 2, 3, and 4, respectively, such that each unit in the population is classified by one of these four index values.

At the endogenous node Y, the counterfactual or potential response variable for unit *u *is denoted by Y_uxz_, where *u *identifies the individual unit and indices x and z specify the X and Z inputs to that unit. Conditional on the individual unit and conditional on the z input, there are four possible patterns of response Y_uxz _to input x: Y_uxz _= 1 regardless of x, Y_uxz _= x, Y_uxz_= 1-x, and Y_uxz _= 0 regardless of x. Therefore, each unit can be fully characterized by one of the 4 × 4 × 4 = 64 possible values of three indices, {ijk}, where index i specifies the Z_ux _response, index j specifies the Y_ux0 _response, and index k specifies the Y_ux1 _response. As an illustration, {123} refers to a unit in which Z will equal 1 regardless of the value taken by X. Under this naturally occurring outcome for Z, Y will equal 1-x. However, if Z were to be manipulated by external intervention to equal 0, then Y would equal x.

In this way, the 64 possible potential response types for individual units in the population are symbolized by {ijk}; i = 1,...,4; j = 1,...,4; k = 1,...,4. We define q_ijk _to be the proportion of type {ijk} in the total population. Furthermore, because X is exogenous, the potential response types occur in these same proportions inboth X = 0 and X = 1 subpopulations. The set of all 64 q_ijk _proportions determines the causal behavior of the population in the context of the three observed variables (X, Y, Z) and potential confounding of the causal effects between them. The values of the 64 q_ijk _proportions, however, are not identified from the 8 observed proportions in the study population: Pr(Y = y, X = x, Z = z); x = 0,1; y = 0,1; z = 0,1.

We make a further simplifying assumption of (strong) monotonicity for the remainder of this paper (see endnote 2). This assumption states that there are no individuals who exhibit preventive effects at either endogenous node. That is, for all units *u *and for z = 0,1 and x = 0,1:

Z_u0 _≤ Z_u1_

Y_u0z _≤ Y_u1z_

Y_ux0 _≤ Y_ux1_

Since the binary values can be arbitrarily coded, the monotonicity of effects can be in any direction (i.e., preventive or causative, since reversing the coding is equivalent to interchangebetween subscript values 2 and 3 and between subscript values 1 and 4). This assumption reduces the number of potential response types in the population from 64 to 18, and may be reasonable on substantive grounds. For example, consider X to be assignment to cholesterol lowering drug cholestyramine versus placebo in the Lipid Research Clinics (LRC) Primary Prevention Trial[24], Z = 1 to be absence of hypercholesterolemia one year after initiation of the cholestyramine, and Y = 1 the absence of coronary heart disease (CHD) at follow-up. In this example, there are no individuals for whom assignment to cholestyramine (X = 1) will cause hypercholesterolemia (Z = 0), nor individuals for whom assignment to cholestyramine or absence of hypercholesterolemia will cause CHD (Y = 0). Note that monotonicity eliminates not only types {3jk}, {i3k} and {ij3}, but also types {i12}, {i14} and {i24}. This is why the assumption reduces the potential outcome patterns not merely to 3 × 3 × 3 = 27, but rather to (3 × 3 × 3)- (3 × 3) = 18. Complete descriptions of the 18 potential outcome types that occur under monotonicity are provided in the first seven columns of Table 1.

**Table 1:** Potential Response Type Characteristics Under Monotonicity Assumption (18 Response Types)

			*a*	*b*	*c*	*d*			
	Response of Y to fixing X to value:		Response of Y to fixing X and Z to values:				Contributes to:		
	
Potential Response Type Representation^†^	X = 1	X = 0	X = 1 Z = 0	X = 0 Z = 0	X = 1 Z = 1	X = 0 Z = 1	Total Effect	Direct Effect in (Z-stratum)	Indirect Effect in (Z-stratum)

{111}	1	1	1	1	1	1			
{141}	1	1	0	0	1	1			
{211}	1	1	1	1	1	1			
{122}	1	0	1	0	1	0	+	+ (0,1)	
{241}	1	0	0	0	1	1	+		+ (0,1)
{222}	1	0	1	0	1	0	+	+ (0,1)	
{411}	1	1	1	1	1	1			
{422}	1	0	1	0	1	0	+	+ (0,1)	
{144}	0	0	0	0	0	0			
{244}	0	0	0	0	0	0			
{441}	0	0	0	0	1	1			
{444}	0	0	0	0	0	0			
{121}*	1	1	1	0	1	1		+ (0)	
{221}*	1	0	1	0	1	1	+	+ (0)	+ (1)
{421}*	1	0	1	0	1	1	+	+ (0)	
{142}*	1	0	0	0	1	0	+	+ (1)	
{242}*	1	0	0	0	1	0	+	+ (1)	+ (0)
{442}*	0	0	0	0	1	0		+ (1)	

* Unit-level interaction (interdependence) present because (*a-b*) ≠ (*c-d*)

^† ^Potential response type representation indices are: 1 = "doomed", 2 = "causal" and 4 = "immune"

Index i of the {ijk}representation specifies the Z_[X = x] _response, index j specifies the Y_[X = x; Z = 0] _response (columns *a *and *b*), and index k specifies the Y_[X = x; Z = 1] _response (columns *c *and *d*).

## 3. Definitions of Causal and Associational Parameters of Interest

The total average causal effect (ACE) of the treatment X in the population is the proportion of all individuals in the population who would experience outcome Y if they were treated, but not if they were untreated, without regard to Z. Given the monotonicity assumption, this effect is the sum of 8 of the 18 potential response type proportions in the population:

ACE_[X→Y] _= average causal effect

= Pr(Y = 1|SET[X = 1]) - Pr(Y = 1|SET[X = 0])

= (q_122 _+ q_241 _+ q_222 _+ q_421 _+ q_422 _+ q_221 _+ q_142 _+ q_242_)

The average causal (controlled) direct effect (ACDE) of the treatment X in the population is the proportion of individuals who would experience outcome Y if they were treated, but not if they were untreated, if Z were forced (SET) to have a specific value z (thus blocking any indirect effects). In general, there is no reason for this effect to take the same value if Z were forced (SET) to 0 as it would take if Z were forced (SET) to 1, and so for binary Z in our DAG there are two distinct average causal direct effects. Given the monotonicity assumption, these effects are the sums of 6 of the 18 potential response type proportions in the population:

ACDE_[X→Y] | SET[Z = 0] _= average causal direct effect for Z forced (SET) to 0

= Pr(Y = 1|SET[X = 1,Z = 0]) - Pr(Y = 1|SET[X = 0,Z = 0])

= (q_122 _+ q_222 _+ q_422 _+ q_121 _+ q_221 _+ q_421_)

ACDE_[X→Y] | SET[Z = 1] _= average causal direct effect for Z forced (SET) to 1

= Pr(Y = 1|SET[X = 1,Z = 1]) - Pr(Y = 1|SET[X = 0,Z = 1])

= (q_122 _+ q_222 _+ q_422 _+ q_142 _+ q_242 _+ q_442_)

A manipulative definition of the total average causal indirect effect, ACIE_[X→Y]_, is not straightforward, and some authors assert that no general definition exists [e.g., [21], p. 165]. The usual interpretations granted to applications of effect decomposition methodology imply that analysts take ACIE_[X→Y] _to mean the proportion of all individuals who would experience outcome Y if they were treated, but not if they were untreated, but only via the pathway in which X has an effect on Z and then Z has an effect on Y. In this causal mechanism, therefore, external intervention to hold Z fixed will prevent X from having any effect on Y. Of the 18 potential response types that exist under the monotonicity assumption, clearly {241} corresponds to this conceptual definition. In units of this type, Z = X. But were Z to be blocked from occurring (i.e., SET to Z = 0) by external intervention, then Y = Z = 0, regardless of X. Alternatively, if Z were to be forced to occur (i.e. SET to Z = 1) by external intervention, then Y = Z = 1. For potential response types {242} and {221}, however, the common-sense meaning of an indirect effect may also apply, depending on the specific intervention applied to Z. Specifically, if the external intervention on the intermediate is SET[Z = 0], then potential response type {242} is an indirect type, whereas if the external intervention on the intermediate is SET[Z = 1], then potential response type {221} is an indirect type (Table 1). This is the ambiguity that has made it difficult to provide a general manipulative definition of the ACIE_[X→Y] _without prohibiting these interacting types, as we do in Section 5.

We can also define the value of the total average causal effect (ACE) of the treatment X on the intermediate covariate Z, which is the proportion of individuals who would experience intermediate Z if they were treated, but not if they were untreated. Given the monotonicity assumption, this effect is the sum of 6 of the 18 potential response type proportions in the population:

ACE_[X→Z] _= average causal effect of X on Z

= Pr(Z = 1|SET[X = 1]) - Pr(Z = 1|SET[X = 0])

= (q_211 _+ q_241 _+ q_222 _+ q_244 _+ q_221 _+ q_242_)

Because the value of Y is determined through the joint effects of X and Z, it is also possible to define the effect of Z on Y as the proportion of individuals who would experience outcome Y if Z were forced (SET) to 1, but not if Z were forced (SET) to 0, conditional on X = x. In general, there is no reason for this effect to take the same value in the X = 0 subpopulation as it does in the X = 1 subpopulation, and so for binary X in our DAG there may be two distinct effects of Z on Y given strata of X. Given the monotonicity assumption, these effects are the sums of 6 of the 18 potential response type proportions in the population:

ACE_[Z→Y] | X = 0 _= Average causal effect of Z on Y in the X = 0 stratum

= Pr(Y = 1|SET[Z = 1], X = 0) - Pr(Y = 1|SET[Z = 0], X = 0)

= (q_141_+ q_241 _+ q_441 _+ q_121 _+ q_221 _+ q_421_)

ACE_[Z→Y] | X = 1 _= Average causal effect of Z on Y in the X = 1 stratum

= Pr(Y = 1|SET[Z = 1], X = 1) - Pr(Y = 1|SET[Z = 0], X = 1)

= (q_141 _+ q_241 _+ q_441 _+ q_142 _+ q_242 _+ q_442_)

Recall that because X is randomized, the potential response type distributionsare independent of X, meaning that the proportions over the total population are the same within the X = 1 and X = 0 subpopulations. We can define ACE_[Z→Y]_, the effect of Z on Y unconditionally, as the proportion of individuals who would experience outcome Y if Z were forced (SET) to 1, but not if Z were forced (SET) to 0, over the entire population. As this is not a stratum-specific quantity, there is only a single value, although this depends on the marginal distribution of X in the population [[21], eq 3.19]. By definition:

ACE_[Z→Y] _= Pr(Y = 1|SET[Z = 1]) - Pr(Y = 1|SET[Z = 0])

= Pr(Y = 1, X = 1|SET[Z = 1]) - Pr(Y = 1, X = 1|SET[Z = 0]) + Pr(Y = 1, X = 0|SET[Z = 1]) - Pr(Y = 1, X = 0|SET[Z = 0])

Given that X is not affected by Z in the specified DAG, this can be re-written as:

(Pr(Y = 1|X = 1,SET[Z = 1]) - Pr(Y = 1|X = 1,SET[Z = 0]) )Pr(X = 1) +

(Pr(Y = 1|X = 0,SET[Z = 1]) - Pr(Y = 1|X = 0,SET[Z = 0]) )Pr(X = 0)

 = Pr(X = 1) ACE_[Z→Y] | X = 1 _+ Pr(X = 0) ACE_[Z→Y] | X = 0_

As shown above, the ACE_[Z→Y] | X = x _terms are each comprised of the sums of 6 of the 18 potential response type proportions in the population, 3 of which are common across the two strata of X and 3 of which are unique to one or the other stratum, so that ACE_[Z→Y] _involves a weighted sum of 9 of the 18 potential response type proportions, with weights dependent upon the marginal distribution of X.

The causal effects defined above are counterfactual, in that they involve hypothetical manipulation of the treatment or intermediate or both. The realized data are the risks that arise in the form of observed proportions in the population. We define R_xz _as the risk (proportion) of Y = 1 among those with X = x and Z = z, i.e., Pr(Y = 1|X = x, Z = z). With binary variables, exogeneity of X and the monotonicity assumption, these observable quantities are related to the latent response type proportions as follows:



The observed risk values R_xz _are used to compute the associational estimates of effect (see endnote 3), as follows:

The risk difference RD_[X→Y] _= R_1• _- R_0• _is the associational estimate of the total average causal effect of X on Y on the additive scale, where R_x• _indicates the risk under X = x collapsed over levels of Z, i.e., R_x• _= Pr(Z = 0|X = x)R_x0 _+ Pr(Z = 1|X = x)R_x1_. Because X is assumed to be randomized and therefore Pr(Z = z|X = x) = Pr(Z = z), RD_[X→Y] _equals the causal RD Pr(Y = 1|SET[X = 1] - Pr(Y = 1|SET[X = 0].

The direct risk difference DRD_[X→Y] | Z = z _= R_1z _- R_0z _is the associational estimate on the additive scale of the average causal direct effect of X on Y within the Z = z stratum. DRD_[X→Y] | Z = z _may be a biased estimate of the analogous causal quantity, i.e., DRD_[X→Y] | Z = z _is not necessarily equal to Pr(Y = 1|SET[X = 1,Z = z]) - Pr(Y = 1|SET[X = 0,Z = z]).

RD_[X→Z] _= Pr(Z = 1|X = 1) - Pr(Z = 1|X = 0) is the associational estimate on the additive scale of the effect of X on Z. Because X is assumed to be randomized, however, this associational estimate equals the analogous causal quantity Pr(Z = 1|SET[X = 1]) - Pr(Z = 1|SET[X = 0]).

RD_[Z→Y] | X = x _= R_x1 _- R_x0 _is the associational estimate on the additive scale of the effect of Z on Y within stratum X = x. Because randomization of X does not imply that the effect of Z on Y is unconfounded, it may be a biased estimate of the analogous causal quantity, i.e., RD_[Z→Y] | X = x _is not necessarily equal to Pr(Y = 1|SET[Z = 1], X = x) - Pr(Y = 1|SET[Z = 0], X = x)

sRD_[Z→Y] _= Pr(X = 1)RD_[Z→Y] | X = 1 _+ Pr(X = 0)RD_[Z→Y] | X = 0 _is the associational estimate on the additive scale of the effect of Z on Y standardized to the distribution of X.

The associational estimate for the indirect effect is generally computed by one of two methods[25]:

1) by subtracting DRD_[X→Y] | Z = z _from RD_[X→Y]_, or

2) by multiplying RD_[X→Z] _by sRD_[Z→Y]_

The first of these methods is the one more commonly applied in epidemiologic research, as represented for example by the expression for "Excess Risk Explained" in Szklo & Nieto[7]. Subtraction of DRD_[X→Y] | Z = z _from RD_[X→Y] _is also recommended in the social sciences methodology literature. For example, Stolzenberg writes: "Once the total and direct effects are calculated, indirect effects may be computed merely by subtracting the direct effect of an antecedent variable from its total effect. This subtraction procedure is applicable both to linear additive models ... and to nonlinear / nonadditive models." [[26] p. 483]. The second method follows from the path analysis rules of Wright [19], and this method also appears in the epidemiologic literature [e.g., [27]].

There will generally be two distinct estimates by the first method above, depending the level chosen for Z. This is a necessary consequence of the manipulative definition of the controlled direct effect, since it involves deactivation of the indirect pathway by preventing variation in Z, and so if Z is to be fixed to a unique value, this value must be specified. In the second method shown above, however, only the two components of the indirect pathway are involved, and so no explicit fixing of Z is specified. This leads to a single estimate of the direct effect, and so the two methods can only be consistent with one another when there is homogeneity of the ACDE over strata of Z (i.e., DRD_[X→Y] | Z = 0 _= DRD_[X→Y] | Z = 1_). The usual regression-based approach for the second method involves the regression of Z on X, followed by the regression of Y on both X and Z, and finally the multiplication of the coefficient estimate for X in the first model by the coefficient estimate for Z in the second model. This conditional estimation of the Z→Y effect in the second model is analogous to taking a weighted average over stratum-specific values as we have done for sRD_[Z→Y]_. Any presumed equivalence of the two approaches shown above by virtue of a homogeneity assumption for the stratum-specific estimates would often be unwarranted, as even under monotonicity it would generally require the additional restrictions that q_142 _= q_242 _= q_442 _= q_121 _= q_221 _= q_421 _= 0 (see endnote 4).

## 4. Absence of Confounding

Since X is randomized in Figure 1, there is no confounding of ACE_[X→Z] _or ACE_[X→Y]. _Furthermore, in our examples we wish to examine the most optimistic scenario in which there is no confounding between Z and Y. We therefore need to formally define exchangeability conditions that imply the absence of confounding. These conditions are a generalization of those provided in Robins and Greenland [[13], eq E1 and E2, p. 149].

We define 4 counterfactual parameters. The first two are the risks of outcome Y among those with X = x and Z = 1 that would have been observed had Z been forced (SET) to take the value 0 rather than the actually occurring value 1.





The second set of counterfactual parameters are the risks of outcome Y among those with X = x and Z = 0 that would have been observed had Z been forced (SET) to take the value 1 rather than the actually occurring value 0.





It is now possible to specify exchangeability conditions that are sufficient to guarantee that there is no confounding, meaning that associational measures and causal effects are equivalent[28]. The four equality conditions that guarantee the absence of confounding between the Z and Y nodes of the DAG are:

R_11|SET[Z = 0] _= R_10_

R_01|SET[Z = 0] _= R_00_

R_10|SET[Z = 1] _= R_11_

R_00|SET[Z = 1] _= R_01_

These conditions assert that the risk that is observed among those with X = x and Z = 0 is the same risk that would have been observed among those with X = x and Z = 1 had Z been forced (SET) from 1 to 0, and that the risk that is observed among those with X = x and Z = 1 is the same risk that would have been observed among those with X = x and Z = 0 had Z been forced (SET) from 0 to 1. The first two sets of exchangeability conditions imply that DRD_[X→Y] | Z = 0 _is unconfounded, whereas the latter two sets of exchangeability conditions imply that DRD_ [X→Y] | Z = 1 _is unconfounded. Under the general scenario in which stratum-specific direct effects may differ, all four conditions are needed to guarantee that there is no confounding in either stratum of Z for any arbitrary choice of effect contrast that may be constructed from the four component risks.

## 5. Example 1: A Restriction that Permits Valid Effect Decomposition

We now impose an additional restriction which, as we will see in Section 6, is necessary for the general validity of the decomposition strategy described above. This restriction is that for no individual in the population may there exist both a causal effect of X on Y and a causal effect of Z on Y. For this condition to hold in general, it must be the case that Z does not modify the effect of X for any unit. This restriction, which can be characterized as the absence of unit-level synergism or interaction, implies homogeneity of the stratum specific direct effects ACDE_[X→Y] | SET[Z = 0] _and ACDE_[X→Y] | SET[Z = 1]_. Under the monotonicity assumption, this requires that 6 of the 18 types, namely {142}, {242}, {442}, {121}, {221}, and {421} be absent from the population. For example, potential response type {242} refers to a unit in which Z will equal x. When X = Z = 1, outcome Y will occur (Y = 1), and when X = Z = 0, outcome Y will not occur (Y = 0), leading to a unit-level casual effect of X on Y equal to (1-0) = 1. However, if Z were to be manipulated by external intervention (SET) to equal 0, then the unit-level effect of X on Y becomes (0- 0) = 0. That is to say, there is no direct effect at this controlled level of Z. In contrast, if Z were to be manipulated by external intervention (SET) to equal 1, the unit-level effect of X on Y remains (1-0) = 1. The direct causal effects of X on Y are heterogeneous for these 6 omitted potential response types because there is unit-level interaction; the value obtained by Y depends not only on the value taken by X, but also on the level to which Z is held by external manipulation. Homogeneity of the stratum-specific direct effects ACDE_[X→Y] | SET[Z = 0] _and ACDE_[X→Y] | SET[Z = 1] _also corresponds to absence of effect measure modification on the additive scale.

When such unit-level synergism is prohibited, then it becomes possible to state an unambiguous definition of the average causal indirect effect (ACIE) of the treatment X in the population as the proportion of all individuals who would experience outcome Y if they were treated, but not if they were untreated, by virtue of the effect that X has on Z and then the effect that Z has on Y. In this mechanism, therefore, external intervention to hold Z fixed will prevent X from having the effect on Y, regardless of the specific value to which Z is SET. Given the restrictions, the average causal indirect effect is merely a single potential response type proportion in the population: ACIE_[X→Y] _= q_241_. The total effect is indeed decomposable into the sum of direct and indirect effects under this restriction, and in the absence of confounding may be estimated without bias. The decomposition is valid because the ACE_[X→Y] _reduces to the sum of only 4 proportions (i.e., q_122_, q_222_, q_422_, and q_241_), since 4 of the previous 8 are restricted to be 0 (i.e., q_221,_q_421, _q_142 _and q_242_). The average causal direct effects (ACDE) of X on Y are the sums of 3 potential response type proportions (rather than 6), which are identical in the two strata: ACDE_[X→Y] | SET[Z = 0] _= ACDE_[X→Y] | SET[Z = 1] _= (q_122 _+ q_222 _+ q_422_). Likewise, ACE_[X→Z] _is the sum of four proportions, ACE_[Z→Y] _= ACE_[Z→Y] | SET[X = 0] _= ACE_[Z→Y] | SET[X = 1] _is the sum of three proportions, and the observed risks R_XZ _are similarly restricted by deleting the prohibited interacting potential response types from the quotients shown above (Table 1).

Consider data arising from a population of unit-level potential response type proportions q_ijk _as shown in Table 2. This population satisfies the restrictions of monotonicity and absence of unit-level synergism. Simple addition of the proportions yields observed risks R_XZ _of R_11 _= 0.9170, R_01 _= 0.5377, R_10 _= 0.6101 and R_00 _= 0.2307. These observed risk values then determine the various associational estimates of effect. The total effect RD_[X→Y] _= (0.8763- 0.3117) = 0.5646. The observed stratum-specific risk differences DRD_[X→Y] | Z = 0 _= DRD_[X→Y] | Z = 1 _= (R_1z _- R_0z_) = 0.3793. Likewise the observed risk difference and stratum-specific risk differences for the effect of Z on Y are also homogeneous, sRD_[Z→Y] _= RD_[Z→Y] | X = 0 _= RD_[Z→Y] | X = 1 _= 0.3070. The observed effect of X on Z, RD_[X→Z] _= 0.6036.

**Table 2:** Example with No Interaction Permitted

Potential Response Type Representation	Prevalence in the Population
{111}	0.0609
{141}	0.0810
{211}	0.1100
{122}	0.0710
{241}	0.1853
{222}	0.2873
{411}	0.0599
{422}	0.0210
{144}	0.0510
{244}	0.0210
{441}	0.0407
{444}	0.0110

Furthermore, the data in this example are constructed such that there is no confounding of the relation between Z and Y. To verify this property, we use the proportions in Table 2 to calculate the values of the counterfactual risks that would be observed under interventions on the intermediate Z. These are: R_11|SET[Z = 0] _= 0.6101; R_01|SET[Z = 0] _= 0.2307; R_10|SET[Z = 1] _= 0.9170; and R_00|SET[Z = 1] _= 0.5377. The absence of confounding is implied by the following set of equalities, the first two of which imply an absence of confounding of DRD_[X→Y] | Z = 0 _and the latter two of which imply an absence of confounding of DRD_[X→Y] | Z = 1_:

R_11|SET[Z = 0] _= R_10 _= 0.6101

R_01|SET[Z = 0] _= R_00 _= 0.2307

R_10|SET[Z = 1] _= R_11 _= 0.9170

R_00|SET[Z = 1] _= R_01 _= 0.5377

In this example, which was constructed to have no confounding and in which potential response types corresponding to unit-level synergism have been eliminated, the associational estimates of the total and direct effects are unbiased. That is, the true total average causal effect equals the observed risk difference (0.5646) and the homogeneous average causal direct effects equal the stratum-specific risk differences (0.3793). It only remains to show that the indirect estimate is valid and that the total effect is decomposable. The true average causal indirect effect in the table is the single potential response type proportion, ACIE_[X→Y] _= (q_241_) = 0.1853. As described above, there are two common approaches for estimating the associational measure of the indirect effect. The first is to subtract DRD_[X→Y] | Z = z _from RD_[X→Y]_, which in this case yields (0.5646- 0.3793) = 0.1853. The second is to multiply RD_[X→Z] _by sRD_[Z→Y]_, which yields (0.6036 × 0.3070) = 0.1853. The estimation of direct and indirect effects and their decomposition from total effects is valid, as will always be the case with this set of assumptions. The justification for this assertion is trivial: this set of assumptions is sufficient to guarantee that the true total ACE is the sum of three ACDE type proportions and one ACIE type proportion, whereas in general, without these restrictions, the ACDE is not constrained to be a subset of the total ACE. We have demonstrated a valid and unbiased estimation of the portion of a total effect that is transmitted through a specified intermediate when there is both absence of confounding and absence of unit-level interaction. We next relax this second constraint in order to demonstrate that the decomposition analysis can then fail.

## 6. Example 2: Removing the Restriction of No Unit-Level Interaction

Now we relax one assumption, the prohibition of unit-level interaction, which was operationalized in Section 5 by requiring that q_142 _= q_242 _= q_121 _= q_221 _= q_421 _= q_442 _= 0. Therefore we have, under the monotonicity restriction alone, 18 potential response types in the population. Consider data arising from a population of unit-level potential response type proportions q_ijk _as shown in Table 3, which conform to this assumption, and additionally are constructed such that there is no confounding. Simple addition of the proportions yields observed risks R_XZ _of R_11 _= 0.9580, R_01 _= 0.3910, R_10 _= 0.4180 and R_00 _= 0.3170. These observed risk values then determine the various associational estimates of effect. The associational estimate of the total ACE is RD_[X→Y] _= (0.8166- 0.3470) = 0.4696. The observed stratum-specific risk differences are no longer constrained to be homogeneous: DRD_[X→Y] | Z = 0 _= (R_10 _- R_00_) = 0.1010 and DRD_[X→Y] | Z = 1 _= (R_11 _- R_01_) = 0.5670. The observed stratum-specific risk differences for the effect of Z on Y similarly need not be homogeneous: RD_[Z→Y] | X = 0 _= 0.0740 and RD_[Z→Y] | X = 1 _= 0.5400. sRD_[Z→Y] _will now depend on the observed marginal distribution of X. If values were assigned with equal probability, then sRD_[Z→Y] _= (0.5 × 0.0740) + (0.5 × 0.5400) = 0.3070. The observed effect of X on Z, RD_[X→Z]_, equals 0.3327.

**Table 3:** Example with Interaction Permitted

Potential Response Type Representation	Prevalence in the Population
{111}	0.1285
{141}	0.0100
{211}	0.1100
{122}	0.0100
{241}	0.0100
{222}	0.0200
{411}	0.0785
{422}	0.0210
{144}	0.0100
{244}	0.0210
{441}	0.0040
{444}	0.0110
{121}	0.0200
{221}	0.0200
{421}	0.0100
{142}	0.2269
{242}	0.1517
{442}	0.1374

To verify that, as in the previous example, the data in this example are unconfounded, the proportions in Table 3 are used to determine the values of the counterfactual risks that would be observed under interventions on the intermediate Z. These are: R_11|SET[Z = 0] _= 0.4180; R_01|SET[Z = 0] _= 0.3170; R_10|SET[Z = 1] _= 0.9580; and R_00|SET[Z = 1] _= 0.3910. The absence of confounding is implied by the following set of equalities, the first two of which imply an absence of confounding of DRD_[X→Y] | Z = 0_and the latter two of which imply an absence of confounding of DRD_[X→Y] | Z = 1 _:

R_11|SET[Z = 0] _= R_10 _= 0.4180

R_01|SET[Z = 0] _= R_00 _= 0.3170

R_10|SET[Z = 1] _= R_11 _= 0.9580

R_00|SET[Z = 1] _= R_01 _= 0.3910

Because X is randomized, the average causal effect of X on Y is identified by the observed associational measure of effect: RD_[X→Y] _= ACE_[X→Y] _= 0.4696. Furthermore, because we have established that there is no confounding, the average causal direct effect of X on Y is identified by the observed associational measure of effect: ACDE_[X→Y] | SET[Z = z] _= DRD_[X→Y] | Z = z_. In this example, ACDE_[X→Y] | SET[Z = 0] _= DRD_[X→Y] | Z = 0 _= 0.1010 and ACDE_[X→Y] | SET[Z = 1] _= DRD_[X→Y] | Z = 1 _= 0.5670. However, in this scenario in which the only assumption we have relaxed is to allow the presence of unit-level synergism, the total average causal effect is no longer decomposable into direct and indirect effects. The average causal indirect effect no longer has single unambiguously true value. If the external manipulation contemplated is to SET[Z = 0], then ACIE_[X→Y] _= (q_241 _+ q_242_) = (0.0100 + 0.1517) = 0.1617. On the other hand, if the external manipulation contemplated is to SET[Z = 1], then ACIE_[X→Y] _= (q_241_+ q_221_) = (0.0100 + 0.0200) = 0.0300.

As described above, there are two common approaches for estimating the associational measure of the indirect effect. The first is to subtract DRD_[X→Y] | Z = z _from RD_[X→Y]_, which in this case yields either (0.4696- 0.5670) = -0.0974 or (0.4696-0.1010) = 0.3686, depending on the stratum of Z, neither of which equals either of the corresponding true values of 0.0300 or 0.1617. The second approach is to multiply RD_[X→Z] _by sRD_[Z→Y]_, which yields (0.3327 × 0.3070) = 0.1021, a value that equals neither of the corresponding true values of 0.0300 or 0.1617, nor is it the weighted average formed from any meaningful set of weights. In this scenario, the estimation of direct and indirect effects and their decomposition from total effects is not valid. It is immediately apparent that once unit-level interaction is permitted, there are potential response types that contribute to the ACDE but which do not contribute to the total ACE, making it incorrect to view the ACDE as a partition of the total ACE. Likewise, there are potential response types that contribute to the ACDE in one stratum of the intermediate, but which contribute to the ACIE in the alternate stratum, making it incorrect to view ACDE and ACIE as adding together to sum to a total effect. Therefore, for the decomposition methodology to be reliable, there must be both absence of confounding and absence of unit-level interaction. Because the absence of unit-level interaction would be difficult to assert with any confidence in a real-world application, the practical utility of decomposition as an analytic strategy is doubtful.

## 7. Discussion

The demonstration above would appear to be somewhat gloomy as regards the potential for analytic epidemiology to identify biologic pathways through the contrast of variously specified statistical models. Indeed, the situation is even more grim than stated above, because even the optimistic scenario in Example 1 (Section 5) relies on the linear causal contrast estimator (i.e., the risk difference). Epidemiologic applications, such as those recommended by Szklo and Nieto [[7], pp. 184–187] nearly always use ratio measures of effects, such as risk ratios, odds ratios and hazard ratios. For ratio contrasts, the total effect is not generally decomposable under any set of causal assumptions. Therefore, the recommended "% Excess Risk Explained", defined as a function of ratio parameters, will never have a causal interpretation and the inference generated will always be ambiguous. In Example 1 (Section 5), for instance, the crude observed RR = 2.81, the Mantel-Haenszel adjusted RR = 2.40, and the Szklo and Nieto "% Excess Risk Explained" therefore equals 22.8%, which does not equal the true proportion of the effect that is relayed though the intermediate, i.e., (ACIE_[X→Y] _/ ACE_[X→Z]_) = (q_241 _/ q_211 _+ q_241_+ q_222 _+ q_244 _+ q_221 _+ q_242_) = (0.1853 / 0.5646) = 32.8%. We note that a valid contrast between total and direct effects for ratio measures of effect was described by Joffe & Colditz[29], but that this does not correspond to a decomposition of effects because the authors did not assume that the ACDE was necessarily a partition of the total ACE.

Even if one steadfastly utilized the risk difference as the causal contrast and justified the no-confounding assumption, in order to reliably decompose the effect, one would still have to believe that there are no units in the population for whom Z and X both affect Y. Under the sharp null hypothesis for the exposure effect, this might be plausible. That is, if X has no effect on Y for any unit, then it follows that there are no units in the population for whom both X and Z have an effect on Y. However an average causal effect equal to the null does not imply this condition. If one were able to assert the no-confounding assumption, then observing that the association parameter is equal to the null would imply that the average causal effect is null, but no observation would imply the absence of unit-level interaction. The observation of heterogeneity would be sufficient to reject the assumption, but the observation of homogeneity would have no implications for this assumption. Nevertheless, as a practical matter, the incidental balancing out of unit-level causal effects leading to homogeneity might be considered unlikely, and therefore as a feasible approximation, the observation of risk difference homogeneity under a substantively defensible assertion of no-confounding might be taken as a setting in which effect decomposition can be attempted with a modicum of credence.

Several previous authors working with latent potential response models have commented on the non-decomposibility of total effects into direct and indirect effects, most notably Robins[30], Robins & Greenland[13] and Pearl [[21], pp. 126–131, 165]. What is perhaps surprising is that although many quantitative sociologists also utilize this same latent outcomes framework [e.g., [20,31,32]], there are to our knowledge no instances of this critique published in the social sciences literature. We find this surprising because effect decomposition is formally embraced in the sociological methodology literature as an essential inferential strategy in the context of structural modeling[25,33]. Indeed, rather than critique this approach, it is strenuously upheld, even for non-linear models[26].

We note that our specification of average causal direct effects in this manuscript corresponds to the *controlled *direct effect, which is to say, the proportion of individuals who would experience outcome Y if they were treated, but not if they were untreated, if Z were to be fixed to have a specific value z (thus blocking any indirect effects). Rather than impose through external intervention a uniform value of Z = z for all units, it is possible to define the average causal direct effect of X on Y that results from fixing Z to the value that would naturally occur under a specific single value of X, for example the unexposed level X = 0. This is referred to by Robins as the "pure direct effect"[34] and by Pearl as the "natural direct effect"[35]. It is noteworthy that this alternate definition does allow for the effect decomposition to hold more generally, and gives rise to additional concepts such as the "total direct effect", which is the difference between the total ACE and the pure (natural) indirect effect. Furthermore, analogously to the controlled direct effects formulation, which leads to as many direct effects as there are levels of intermediate Z, the pure (natural) direct effects formulation leads to as many direct effects as there are levels of exposure X.

Although it allows for decomposition without the assumption of no unit-level interaction, the approach involving pure (natural) direct and indirect effects has a substantial deficiency. The exchangeability conditions shown above (Section 4) characterize confounding in relation to hypothetical but defined manipulations of the target population. That is, X and Z are controlled to specific values. Because the pure (natural) direct and indirect effects are defined based on intermediate Z being manipulated to an unobserved value that it would have taken under an exposure X value that may not have occurred, the exact nature of this intervention remains obscure. Because the hypothetical manipulation cannot be specified, the decomposed effects no longer have any possible relevance to a specific public health intervention or policy [[34], Section 3].

For example, if one were to estimate that the pure (natural) indirect effect through intermediate Z equals 50% of the total effect, one could not infer that 50% of the outcomes attributable to the exposure could be prevented by blocking Z from occurring. Controlled direct effects can be used to make statements such as "The effect that postmenopausal hormone therapy would have on breast cancer risk if we were to persuade every woman to receive a screening mammography is ...." No similarly practical statement could be made for a pure (natural) direct effect, however, which would correspond to something like "The effect that postmenopausal hormone therapy would have on breast cancer risk if every woman were to engage in the screening mammography behavior that she would have exhibited under the absence of treatment is ...." This latter statement obviously has no clear public health policy implications, since it requires a policy of fixing the intermediate to different values, some of which are unobserved (e.g., the screening behavior that a woman taking postmenopausal hormone therapy would have experienced had she not taken hormone therapy).

In summary, the ubiquitous strategy of adjusting for one or more putative causal intermediates in order to estimate the portion of the effect that it mediated by this pathway, in epidemiology and in other fields, lacks a reliable foundation. There are highly constrained sets of assumptions which allow this strategy to be valid, but it is often difficult to know when, if ever, these assumptions are approximately satisfied. Previous critiques have focused on confounding between the intermediate and the outcome, but we show that even when there is no confounding, the total causal effect of treatment is not generally decomposable into direct and indirect effects. Valid estimation of the direct or indirect effects, or of the proportion of the total effect that is due to an intermediate variable, requires not only the assumption of no confounding, but also the assumed absence of unit-level synergism, the latter of which may be particularly difficult to assert in a real-world analysis. Furthermore, even under these conditions, the decomposition is only valid for the difference contrast as the measure of causal effect, not for ratio measures of effect such as risk ratios, rate ratios, hazard ratios or odds ratios. In circumstances when it is possible to estimate (controlled) average causal direct effects, these should not generally be interpreted as portions of the total average causal effect, nor should they generally be used to make any statement about the proportion of the effect that is attributable to the measured intermediate variable.

## List of Abbreviations Used

ACE average causal effect

ACDE average causal (controlled) direct effect

ACIE average causal (controlled) indirect effect

CHD coronary heart disease

DAG directed acyclic graph

DRD direct risk difference

LRC Lipid Research Clinics

RD risk difference

RR relative risk or risk ratio

sRD standardized risk difference

SUTVA stable-unit-treatment-value assumption

## Competing Interests

The authors declare that they have no competing interests.

## Endnotes

1. Because of the assumption of zero sampling error "proportions" (in the observed sample) and "probabilities" (in the source population)are interchangeable.

2. The characterization of this monotonicity assumption as *strong *is intended to distinguish it from a weaker stochastic monotonicity assumption that may be defined:

Pr(Z_0 _= 1) ≤ Pr(Z_1 _= 1)

Pr(Y_0z _= 1) ≤ Pr(Y_1z _= 1); z = 0,1

Pr(Y_x0 _= 1) ≤ Pr(Y_x1 _= 1); x = 0,1

where Z_x _is the random variable representing the potential response at Z to SET[X = x], and Y_xz _is the random variable representing the potential response at Y to SET[X = x, Z = z].

3. Associational estimates are obtained from contrasts in the observed data, rather than being estimates of what would pertain under the hypothetical manipulation that is indicated by a SET operation.

4. Strictly, equivalence of the two approaches for specifying the indirect effect requires merely that (q_142 _+ q_242 _+ q_442_) = (q_121 _+ q_221 _+ q_421_), but this incidental cancellation would be difficult to anticipate, whereas the absence of some potential outcomes types is a more plausible form of background knowledge that an investigator could bring to the analysis.

## Authors' Contributions

JSK led the writing, but all three authors contributed heavily to editing and revision. RFM devised the notational system and the numeric examples.
